# *Sophora moocroftiana* seeds ethanol extract regulates NLRP3 inflammasome activation and pyroptosis via ROS/TXNIP pathway to amelioration of NAFLD *in vitro* and *in vivo*

**DOI:** 10.3389/fphar.2025.1622178

**Published:** 2025-08-25

**Authors:** Ruiying Yuan, Mingan Gui, Shan Huang, Yangzhen Ciren, Dongzhi Zhuoma, Guibin Yang, Wendong Zhang, Sicen Wang, Bin Li

**Affiliations:** ^1^ Department of Medicament, College of Medicine, Xizang University, Lhasa, China; ^2^ Department of Pharmacy, Key Laboratory of Pharmaceutical Research for Metabolic Diseases, Qingdao University of Science and Technology, Qingdao, China; ^3^ School of Pharmacy, Health Science Centery, Shaanxi Engineering Research Center of Cardiovascular Drugs Screening and Analysis, Xi’an Jiaotong University, Xi’an, China

**Keywords:** Sophora moocroftiana seeds, non-alcoholic fatty liver disease, pyroptosis, NLRP3 inflammasome, ROS/TXNIP pathway

## Abstract

**Background:**

*Sophora moorcroftiana* (Benth.) Baker is a perennial shrub endemic to the Tibetan Plateau. Its seeds are traditional Tibetan medicine for treating jaundice, hepatitis, purulent tonsillitis, diphtheria, and parasitosis.

**Aim of the study:**

The aim of this research is to explore the possible processes by which the 70% ethanol extract of *S. moocroftiana* seeds (SMS) imparts healing effects in NAFLD what is done by simulating the disease state of NAFLD *via* the creation of a lipid accumulation model in HepG2 cells induced by FFA, and a NAFLD model in C57BL/6J mice induced by HFD.

**Materials and methods:**

The experiment used HFD fed C57BL/6J mice to establish a NAFLD model, which was divided into a control group, HFD model group, different doses of SMS groups, and a Silymarin group. Were used in the experiment. SMS could improve lipid accumulation, reduce oxidative stress and inflammatory response, which confirmed the improvement effect of SMS on NAFLD. The protective mechanism of SMS on HepG2 cell lipid accumulation model was further verified by qRT-PCR, WB, ELISA, and other methods.

**Results:**

*In vitro* and *in vivo* experiments showed that SMS could inhibit ROS and TXNIP dissociation, regulate NLRP3/caspase 1/GSDMD pathway to inhibit pyroptosis, and reduce the release of inflammatory factors such as TNF-α, IL-6, IL-18, and IL-1β, thereby improving the symptoms of lipid accumulation and oxidative stress. These results suggest that SMS can improve NAFLD *via* regulating pyroptosis.

**Conclusion:**

SMS has promising therapeutic potential in NAFLD management by inhibiting the activation of the NLRP3 inflammasome and pyroptosis via ROS/TXNIP pathway in hepatocytes.

## Graphical Abstract

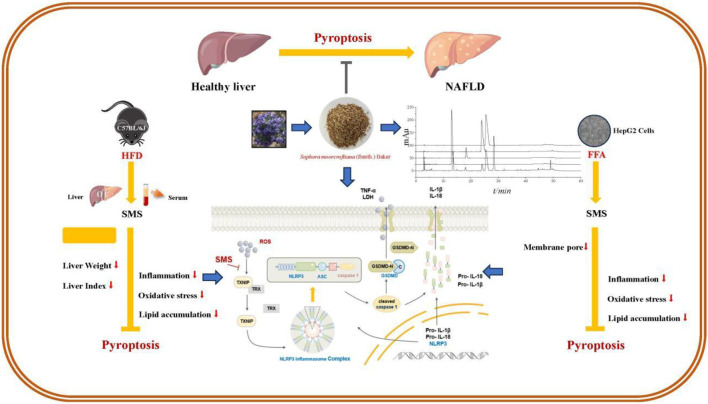

## 1 Introduction

Non-alcoholic fatty liver disease (NAFLD) represents a complex spectrum of hepatic disorders, ranging from excessive fat deposition in the liver to advanced stages involving significant hepatocyte injury, inflammation, and fibrosis. NAFLD is often accompanied by oxidative stress and inflammatory response (Serviddio et al., 2013; Arrese et al., 2016). Reactive oxygen species (ROS) generated intracellularly constitute the primary source of oxidative stress. The overproduction of ROS hampers the efficacy of antioxidative defense mechanisms in NAFLD (Anania et al., 2018). An imbalance between ROS production and the body’s antioxidant defense mechanisms can lead to oxidative stress. In NAFLD, excessive lipid accumulation in liver cells leads to increased oxidation of fatty acids in mitochondria and peroxisomes, damaging cellular components, including lipids and proteins. ROS also activates pro-inflammatory signaling pathways that further promote the occurrence and development of oxidative stress in NAFLD (Chen et al., 2020). ROS have long been recognized as pivotal contributors to pyroptosis, apoptosis, and necroptosis, contributing to various forms of cell demise (Farzanegi et al., 2019).

Pyroptosis, delineated as a newly acknowledged form of regulated cell death, is characterized by cytoplasmic membrane pore formation, cellular swelling, and rupture, culminating in the release of pro-inflammatory cytokines interleukin-18 (IL-18) and interleukin-1β (IL-1β) (Wang et al., 2020). Pyroptosis of hepatocytes and the NOD-like receptor thermal protein domain associated protein 3 (NLRP3) inflammasome it releases play a key role in liver inflammation and fibrosis. It was found in NAFLD patients that after activation of the NLRP3 inflammasome, hepatocytes pyroptosis occurs and releases inflammasome protein, which further drives inflammation and fibrosis reduce liver injury (Gaul et al., 2021). ROS leads to leading to the dissociation of thioredoxin-interacting protein (TXNIP) from the TXNIP-TRX complex. (Zhou et al., 2010). TXNIP is a major cellular antioxidant and anti-apoptotic system, which binds to thioredoxin (TRX) and maintains an inactive state (Zhang et al., 2015). TXNIP is crucial for the activation of NLRP3 inflammasome, and the dissociation of TXNIP from TRX activates NLRP3 (Zhou et al., 2010). By inhibiting the expression of NLRP3, the occurrence of pyroptosis can be suppressed, thus alleviating the inflammatory response of NAFLD (Ruan et al., 2021). Gasdermin D (GSDMD) is the execution protein that drives pyroptosis, and the activation of GSDMD is closely related to the activation of NLRP3 inflammasome (Zheng and Kanneganti, 2020; Yan et al., 2021). NLRP3 can interact with apoptosis associated speck-like protein containing a caspase recruitment domain (ASC) to cleave Caspase-1 and form mature cleaved caspase-1. GSDMD is specifically cleaved by caspase-1, which can bind in the cellular membrane and form extensive pores, thereby triggering pyroptosis (Shi et al., 2015; Shi et al., 2017), as well as releasing pro-inflammatory cytokines such as IL-1β (Liu et al., 2016). Recent studies have shown that pyroptosis can lead to acute or chronic liver injury (Zhao et al., 2022) and reducing of hepatocyte pyroptosis has a beneficial effect on NAFLD (Ezquerro et al., 2018).

*Sophora moorcroftiana* (Benth.) Baker, a perennial shrub native to the Qinghai-Tibet Plateau, thrives at elevations between 2,900 and 4,100 m (Liu et al., 2006). According to Tibetan medicinal traditions, the seeds of *S. moorcroftiana* are employed in the treatment of jaundice-type hepatitis and are recognized for their anti-inflammatory, antibacterial, detoxifying properties, and other advantageous effects (Luo Y. P. et al., 2018; Hu et al., 2022), as well as in combating echinococcosis (Zhang F. et al., 2018). Previous phytochemical investigations have identified alkaloids, flavonoids, and polysaccharides in this species (Zhang Y. et al., 2018; Luo Y. et al., 2018; Shirataki et al., 1988). Notably, oxymatrine has shown efficacy in reducing lipid accumulation, while matrine addresses mitochondrial dysfunction, accumulation disorders, and inflammation, thereby ameliorating NAFLD (Gao et al., 2018; Zhang et al., 2020). In our prior *in vivo* and *in vitro* research, the ethanol extract of *S. moorcroftiana* seeds demonstrated substantial hepatoprotective properties (Yuan et al., 2022). Despite these findings, investigations into its potential for improving NAFLD remain scarce.

This study aims to investigate how *S*. *moorcroftian*a seeds ameliorate NAFLD by modulating the TXNIP/NLRP3/GSDMD *via* pyroptosis pathway, thereby providing a potential therapeutic strategy for NAFLD treatment.

## 2 Materials and methods

### 2.1 Chemicals and reagents

Matrine (≥98% purity), Sophocarpine (≥98%), Oxysophocarpine (≥98%), and Oxymatrine (≥98%) were obtained from Chengdu Must Bio-Technology Co., Ltd. (Chengdu, China), while the remaining chemicals were sourced from Sigma Chemical Co. (St. Louis, United States). HepG2 cells were cultured in a specialized medium obtained from Procell Life Science & Technology Co., Ltd. (Wuhan, China). Primary antibodies, including mouse/rabbit anti-TXNIP (DF7506), anti-NLRP3 (BF8029), anti-Caspase 1 (AF5418), anti-cleaved Caspase-1 (AF4005), anti-GSDMD (AF4012), anti-GSDMD-N (DF13758), anti-IL1β (BF8021), and anti-β-actin (AF7018), were procured from Affinity Biopharmaceutical Co., Ltd. (Nanjing, China). Horseradish peroxidase-conjugated goat immunoglobulin G (IgG) (ab150113) and rabbit anti-mouse IgG (ab150077) secondary antibodies were purchased from Abcam Plc. (Cambridge, United Kingdom). Various kits used in the experiment, including triglyceride (TG), total cholesterol (TC), low-density lipoprotein cholesterol (LDL-C), high-density lipoprotein cholesterol (HDL-C), superoxide dismutase (SOD), malondialdehyde (MDA), catalase (CAT), glutathione peroxidase (GSH-PX), aspartate aminotransferase (AST), alanine aminotransferase (ALT), alkaline phosphatase (AKP) was acquired from Jiancheng Biotechnology Science, Inc. (Nanjing, China). Radioimmunoprecipitation lysis buffer (RIPA), phenylmethanesulfonyl fluoride (PMSF), and Bradford protein assay kits were purchased from Solaibo Technology Co., Ltd. (Beijing, China). The TRIeasy Plus Total RNA Kit reagents were obtained from Enzyme-linked Biotechnology Co., Ltd. (Shanghai, China), while BeyoFast SYBR Green One-Step qRT-PCR Kits were sourced from Beyotime Biotechnology (Shanghai, China). The levels of IL-1β, IL-18, IL-6, and tumor necrosis factor-α (TNF-α) were measured using enzyme-linked immunosorbent assay (ELISA) kits from Boster Biological Technology Co. Ltd. (Wuhan, China).

### 2.2 Extraction of *S. moocroftiana* seeds

*S. moorcroftiana* seeds were purchased from Lhasa in March of 2022 and their authenticity was confirmed by Professor Zhuoma Dongzhi of the College of Medicine at Tibet University in Lhasa, Tibet, China. A sample of the seeds, for which the voucher specimen (No. utibet-1539) was stored in the Herbarium of Herbarium of Department of Medicament, College of Medicine, Tibet University, Lhasa, China. The seeds, which weighed 5,005.1 g, were pulverized and extracted with 70% ethanol by refluxing. After filtering and concentrating under reduced pressure, 1,023.1 g of extract was obtained (20.44% yield). The extract was preserved in the Component Bank of Tibetan Medicine in Lhasa (No. CBTM-E396), Tibet, China for potential future research purposes.

### 2.3 High performance liquid chromatography (HPLC) analysis

Quantitative analysis of the plant metabolites of SMS was carried out utilizing HPLC. In this process, methanol was used to dissolve standard substances containing Matrine, Sophocarpine, Oxysophocarpine, and Oxymatrine at concentrations of 511.0, 170.0, 365.0, and 1,250.5 μg/mL, respectively. SMS at a concentration of 3.76 mg/mL, was also dissolved in methanol. The separation was performed on an Agilent 5 HC-C18 column (250 mm × 4.6 mm, particle size 5 μm) using an LC-2010A HT HPLC system (Shimadzu Corporation, Kyoto, Japan) with HPLC and detection at a wavelength of 210 nm. The mobile phase consisted of acetonitrile (A) and a 0.1% aqueous solution of phosphoric acid (B) with a linear gradient elution: 0–20 min with 3% A, 20–45 min with 3%–15% A, and 45–60 min with 15% A. The flow rate was maintained at 1.0 mL/min, and a 15-min column wash was conducted between injections. After injection, the column was equilibrated with 3% A for 30 min. The column temperature was set at 20 °C, and the injection volume was 5 μL. The identification of plant metabolites in SMS was achieved by comparing the retention times of corresponding standard compounds.

### 2.4 Experimental animals

Male C57BL/6J mice (weighing 20 ± 2 g) were purchased from the Qingdao Institute for Food and Drug Control (approval number: SYXK (Lu) 2022 0816). These mice were acclimatized to controlled environmental conditions (temperature: 25 °C ± 2 °C, relative humidity: 55% ± 5%, and a 12/12 h light/dark cycle) for a period of 7 days, during which they had unrestricted access to food and water. The experimental protocols were ethically approved by the Institutional Animal Care and Use Committee of Qingdao University of Science and Technology (approval number: ACQUST-2020-053).

### 2.5 Induction of NAFLD mice and experimental design

The mice were divided into two groups: a control group (CON) with 10 mice and a model group with 50 mice. The CON group received a normal-fat diet comprising 30% wheat, 30% corn, 10% fish, 3% bone meal, and 7% soybean meal. In contrast, the model group was fed a high-fat diet composed of 80% NFD and 20% high-fat feed (containing 3% cholesterol, 7% lard, and egg yolk powder) for 8 weeks. Serum levels of TC, TG, ALT, and AST confirmed successful model group establishment using reagent kits. Subsequently, the model group was further divided into five subgroups (n = 10 each): the high-fat diet model group (HFD), a positive control group (administered Silymarin at 100 mg/kg), and low, medium, and high dosage groups (L: 52 mg/kg, M: 104 mg/kg, and H:208 mg/kg) of the SMS. While the CON and HFD groups received normal saline, the treatment group needed to be given SMS by intragastric administration at a fixed dose every day for 8 weeks. After a 12-h fasting period, blood samples were collected from the orbital vein and centrifuged to obtain serum samples. Liver samples were dissected, weighed, and half of them were stored at −80 °C, while the remaining half was preserved in a 4% polyformaldehyde solution for subsequent experiments.

### 2.6 Hematoxylin eosin (H&E) staining

The liver tissues were fixed in polyformaldehyde, dehydrated, paraffin-embedded, and sectioned to a thickness of 5 μm. Subsequently, the sections underwent H&E staining. Observations were made on these sections under a microscope (Nikon, Tokyo, Japan), and random areas were chosen for photography.

### 2.7 Immunohistochemistry assay

Immunohistochemical staining was conducted using 5 μm paraffin sections that were dewaxed in xylene. The sections were dehydrated and incubated with 3% H_2_O_2_ for 10 min, followed by washing with PBS. After blocking with PBS containing 5% fetal bovine serum (FBS), the sections were incubated overnight at 4 °C with primary antibodies against TXNIP, NLRP3, GSDMD, and IL-6 (1:200). This was followed by incubation with goat anti-rabbit IgG (H + L) HRP at 37 °C. After incubation with Streptavidin-HRP, 2,2′-Dimethylbenzidine (DAB) staining was performed, followed by hematoxylin counterstaining. Randomly selected fields were photographed at ×200 magnification using a light microscope (Nikon, Tokyo, Japan).

### 2.8 Cell culture and treatment

HepG2 cells, obtained from Procell Life Science & Technology Co., Ltd. (Wuhan, China), were cultured in minimum essential medium (MEM) with NEAA supplemented with 10% FBS and 1% penicillin/streptomycin (P/S). The cells were maintained at 37 °C in a 5% CO_2_ conditions, with sub-culturing performed every 2–3 days. Initially, the HepG2 cells were exposed to varying concentrations of SMS (12.5, 25, 50, and 100 μg/mL) for 24 h. Then, the cells received an additional treatment with free fatty acid (FFA, 1.0 mM) for an extra 24 h.

### 2.9 Cell viability

Cell viability was evaluated using the MTT assay. HepG2 cells were cultured in a 96-well plate at a concentration of 6 × 10^5 cells/mL (100 μL). The cells were exposed to SMS either alone or at varying concentrations (12.5, 25, 50, and 100 μg/mL), for 24 h. Additionally, the cells received an extra treatment with FFA (Sodium oleate (OA) and sodium palmitate (PA) were dissolved in a 20% bovine serum albumin (BSA) solution and mixed at a ratio of 2:1, 1.0 mM) for an additional 24 h. Subsequently, a solution of 3-[4,5-dimethylthiazol-2-yl]-2,5-diphenyltetrazolium bromide (MTT, 2.5 mg/mL, 50 μL) was added to each well. After a 4-h incubation, formazan crystals were dissolved in Dimethyl sulfoxide (DMSO), and the optical density (OD) was measured at a wavelength of 490 nm. The initial OD was recorded at the time of compound addition.

### 2.10 ROS fluorescence probe detection

HepG2 cells were cultured in a 6-well plate at a concentration of 6 × 10^5 cells/mL (1 mL). Detection of ROS was conducted utilizing an ROS Assay Kit, following the manufacturer’s protocol. Diluted DCFH-DA (1:1,000) was added to each well, and the cells were incubated at 37 °C for 20 min in a cell culture incubator. After incubation, the cells were wished thrice with cell culture medium, and fluorescence was visualized using a laser confocal microscope (Olympus Corporation DP74, Tokyo, Japan).

### 2.11 Oil red O staining

To evaluate lipid accumulation in liver tissue and HepG2 cells, liver sections underwent fixation using 4% formaldehyde and subsequent staining with Oil Red O. After isopropanol washing, the sections were counterstained with hematoxylin and examined using a microscope (Olympus Corporation DP74, Tokyo, Japan). HepG2 cells were stained using the Oil Red O kit designed for cultured cells (Solarbio, Beijing, China). Random areas were selected for photographic documentation via light microscopy (Olympus BX53, Tokyo, Japan).

### 2.12 Western blot (WB) analysis

Protein specimens were initially gathered from HepG2 cells and the liver tissues of mice. The isolation procedure utilized RIPA buffer, supplemented with 1% PMSF. The Bradford protein assay kit was used to quantify the protein quantities. Following this, protein samples of equal amounts were differentiated using 10%–15% Sodium dodecyl sulfate-polyacrylamide gel electrophoresis (SDS-PAGE). These were then transferred onto NC membranes. The membranes were treated with 5% skim milk in TBST for a duration of 2 h at room temperature. Primary antibodies specific were applied to the membranes for 1.5 h at room temperature. After washing the membranes thrice with TBST, secondary antibodies were introduced for 1 h at room temperature. The protein bands were visualized using an Enhanced chemiluminescence kit (ECL). A ChemiDoc image analyzer (Tanon 4,600, Shanghai, China) was used to ascertain protein concentrations, and ImageJ software (NIH, Bethesda, MD, United States) was utilized for image analysis and manipulation.

### 2.13 RNA isolation and quantitative real-time polymerase chain reaction (qRT-PCR)

The process of isolating RNA from HepG2 cells utilized the TRIeasy Plus Total RNA Kit. This was followed by reverse transcription and qRT-PCR, facilitated by the BeyoFast SYBR Green One-Step qRT-PCR Kit, to measure the mRNA levels of TNF-α, IL-6, IL-18, IL-1β, and TXNIP. β-actin mRNA served as a control in these experiments. The entire procedure was repeated three times, and the Livak method was used to assess mRNA expression levels. ΔCt represents the difference between the Ct value of the target gene and the Ct value of the reference gene. ΔΔCt​ is the difference between the ΔCt values of the experimental groups and those of the control group. The primer sequences are provided in Table 1.

$$mRNA\;\;expression\;levels=2^{-\Delta \Delta C_{t}}\left(Livak\;\;method\right)$$

**TABLE 1:** List of primers for qRT-PCR.

Target	Primer sequences (forward)	Primer sequences (reverse)
IL-6	ACC​ACT​CCC​AAC​AGA​CCT​GTC​T	CAG​ATT​GTT​TTC​TGC​AAG​TGC​AT
TNF-α	ACA​AGG​CTG​CCC​CGA​CTA​C	TGG​GCT​CAT​ACC​AGG​GTT​TG
IL-18	GAC​TCT​TGC​GTC​AAC​TTC​AAG​G	CAG​GCT​GTC​TTT​TGT​CAA​CGA
IL-1β	CTT​TCC​CGT​GGA​CCT​TCC​A	CTC​GGA​GCC​TGT​AGT​GCA​GTT
TXNIP	AGC​CAA​CTC​AAG​AGG​CAA​AG	GAG​ACT​GAC​ACA​CGT​CCA​TC
β-actin	GGC​TGT​ATT​CCC​CTC​CAT​CG	CCA​GTT​GGT​AAC​AAT​GCC​ATG​T

### 2.14 Biochemical analysis

The lipid deposition was detected by measuring the levels of TG, TC, LDL-C, HDL-C in both serum and HepG2 cells using a reagent kit. Oxidative stress was detected by measuring the levels of SOD, CAT, GSH-PX and the activity of MDA in both serum and HepG2 cells using a reagent kit. Liver function was assessed by measuring the levels of AST, ALT, and AKP in serum using reagent kits. The levels of IL-1β and IL-18 in serum homogenates and HepG2 cells, as well as IL-6 and TNF-α in liver tissues were detected using ELISA kits. Additionally, the mRNA levels of TNF-α, IL-6, IL-18, and IL-1β in HepG2 cells were determined using qRT-PCR, as mentioned earlier.

### 2.15 Immunofluorescence assay

The HepG2 cells, after appropriate preparation, were rinsed with PBS and then fixed with 4% paraformaldehyde for 30 min. Subsequently, the cells were treated with 0.1% Triton X-100 for 10 min and then blocked using a solution of PBS containing 5% FBS at ambient temperature for another 30 min. The primary antibody was applied and allowed to incubate overnight at 4 °C. This was followed by an incubation period with FITC-conjugated goat anti-mouse IgG or rabbit anti-rabbit IgG secondary antibody at room temperature for 1 h, in conjunction with a 5-min incubation with DAPI. The immunostaining was then observed using a fluorescence microscope (Olympus Corporation DP74, Tokyo, Japan).

### 2.16 Scanning electron microscopy (SEM)

The HepG2 cells, after appropriate preparation, were washed with PBS to remove the culture medium and then fixed with 5% glutaraldehyde for 1 h at room temperature. Post the glutaraldehyde exposure, gradient concentrations of ethanol were successively dehydrated for 15 min. The samples were then subjected to a 12-h freeze-drying period in a vacuum freeze-dryer. Pretreatment steps were performed on the cells using ion sputter coating technology to stabilize the cell surface and mitigate charge effects. The structure of the cell membrane was then examined by SEM.

### 2.17 Cell transfection with small interfering RNA (siRNA) targeting TXNIP

In order to investigate the relationship between TXNIP and pyroptosis, HepG2 cells were transfected with TXNIP-siRNA, procured from GenePharma Co., Ltd. (Suzhou, China), in accordance with the instructions provided by the manufacturer. The transfection process was initiated when the cell density was within the range of 60%–80%. After transfection, the cells were incubated at 37 °C for a duration of 4–6 h, following which they were transferred to complete culture medium. The expression of mRNA was examined within a period of 24–72 h, while protein expression was analyzed within a timeframe of 48–96 h.

### 2.18 Statistical analysis

The experimental data underwent analysis using GraphPad Prism 7 software, developed by GraphPad Software in San Diego, CA, United States of America. The data were presented as mean ± Standard deviation (SD). For continuous data, a one-way ANOVA was applied, followed by Tukey’s *post hoc* test for inter-group comparisons. Statistical significance is indicated as ^*^ *P* < 0.05, ^**^ *P* < 0.01, ^#^ *P* < 0.05, ^##^ *P* < 0.01 and ^§^ *P* < 0.05, ^§§^ *P* < 0.01.

## 3 Results

### 3.1 Quantitative analysis of the four ingredients in SMS identified by HPLC

**FIGURE 1:**
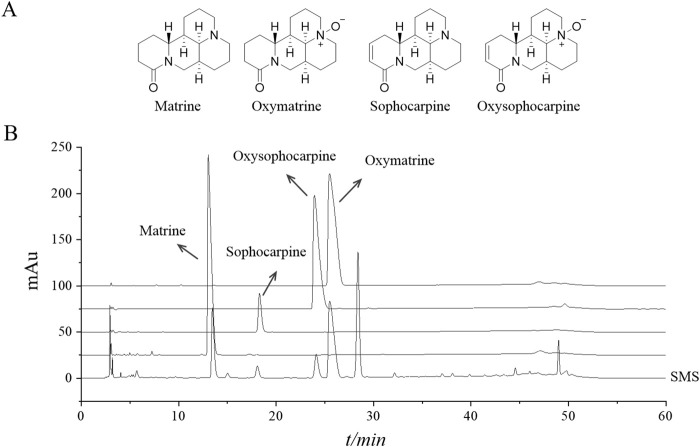
The representative ingredients in SMS were analyzed by HPLC. **(A)** The chemical structure of Matrine, Sophocarpine, Oxysophocarpine and Oxymatrine and **(B)** chromatograms of four plant metabolites in reference substances and SMS.

The HPLC spectra representative of SMS are shown in Figure 1B. The primary constituents of SMS are detailed in Table 2. The chemical formulas for Matrine, Sophocarpine, Oxysophocarpine, and Oxymatrine are illustrated in Figure 1. As per the regression equation of the curve, the concentrations of Matrine, Sophocarpine, Oxysophocarpine, and Oxymatrine in *S. moorcroftiana* seeds are 8.26, 1.41, 3.59, and 45.89 mg/g, respectively.

**TABLE 2:** Preliminary identification of compounds and content determination in SMS.

Compound name	Molecular formula	R.T. (min)	Curve regression equations	R^2^	Linear range (μg/mL)	Content (mg/g)
Matrine	C_15_H_24_N_2_O	13.04	Y = 9,250,264.11 X - 27,270.73	0.9996	51.1–511.0	8.26
Sophocarpine	C_15_H_22_N_2_O	18.29	Y = 12,170,176.15 X - 21,157.64	0.9995	17.0–170.0	1.41
Oxysophocarpine	C_15_H_22_N_2_O_2_	23.93	Y = 10,639,731.88 X - 28,282.10	0.9996	36.5–365.0	3.59
Oxymatrine	C_15_H_24_N_2_O_2_	25.49	Y = 3,986,402.89 X - 26,149.87	0.9996	125.1–1,250.5	45.89

### 3.2 SMS restored on liver injury of NAFLD in HFD fed C57BL/6J mice

**FIGURE 2:**
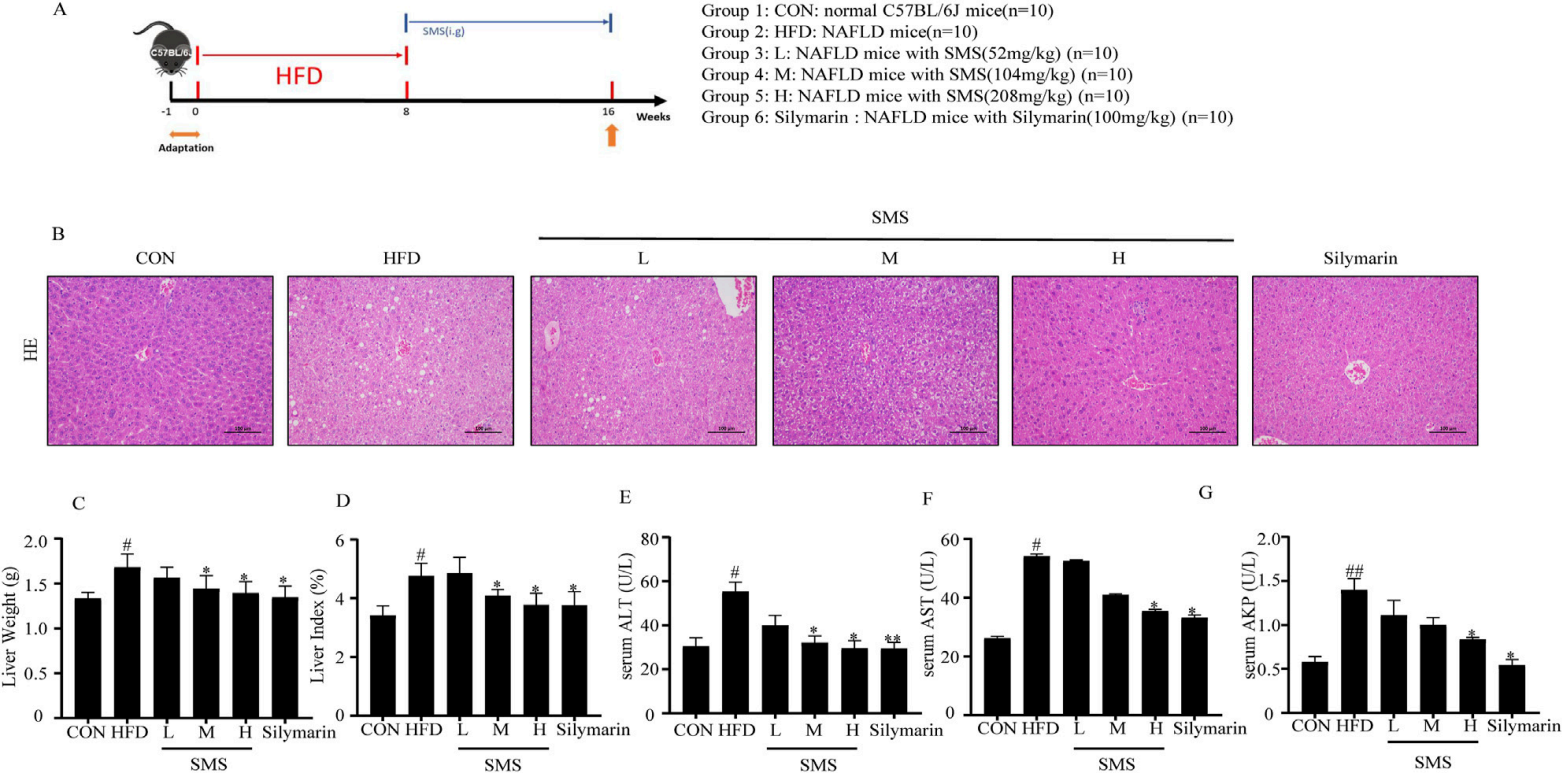
Effect of SMS on hepatic injury of NAFLD in HFD-fed C57BL/6J mice. **(A)** Experimental procedure of mice. **(B)** H&E staining were performed on liver tissue sections of each mice and a representative image of one mice per group has been displayed (200 ×), The levels of **(C)** liver weight and **(D)** index. Serum **(E)** ALT, **(F)** AST and **(G)** AKP were detected by reagent kits. Silymarin (100 mg/kg) was used as the positive control. Results are mean ± SD (n = 10). ^#^ *P* < 0.05, ^##^ *P* < 0.01 vs. CON group; ^*^ *P* < 0.05, ^**^ *P* < 0.01 vs. HFD group.

The effect of SMS on improving accumulation of lipids was confirmed by evaluating liver histopathology. Liver histopathology revealed significant hepatic injury in the HFD group compared to the CON group. However, H&E staining indicated orderly ordering of hepatocytes and reduced fat infiltration after SMS treatment (Figure 2B). After 8 weeks of treatment, a significant decrease in liver weight and index was observed in the SMS group (Figures 2C,D). Treatment with SMS also reduced the levels of ALT, AST, and AKP compared to the FFA group in a dose-dependent manner,The levels of ALT, AST and AKP in L, M and H groups were significantly affected (Figures 2E–G). Collectively, these findings suggest that SMS exhibits a protective effect against liver injury of NAFLD *in vivo*.

### 3.3 SMS alleviate lipid deposition of NAFLD in HFD fed C57BL/6J mice

**FIGURE 3:**
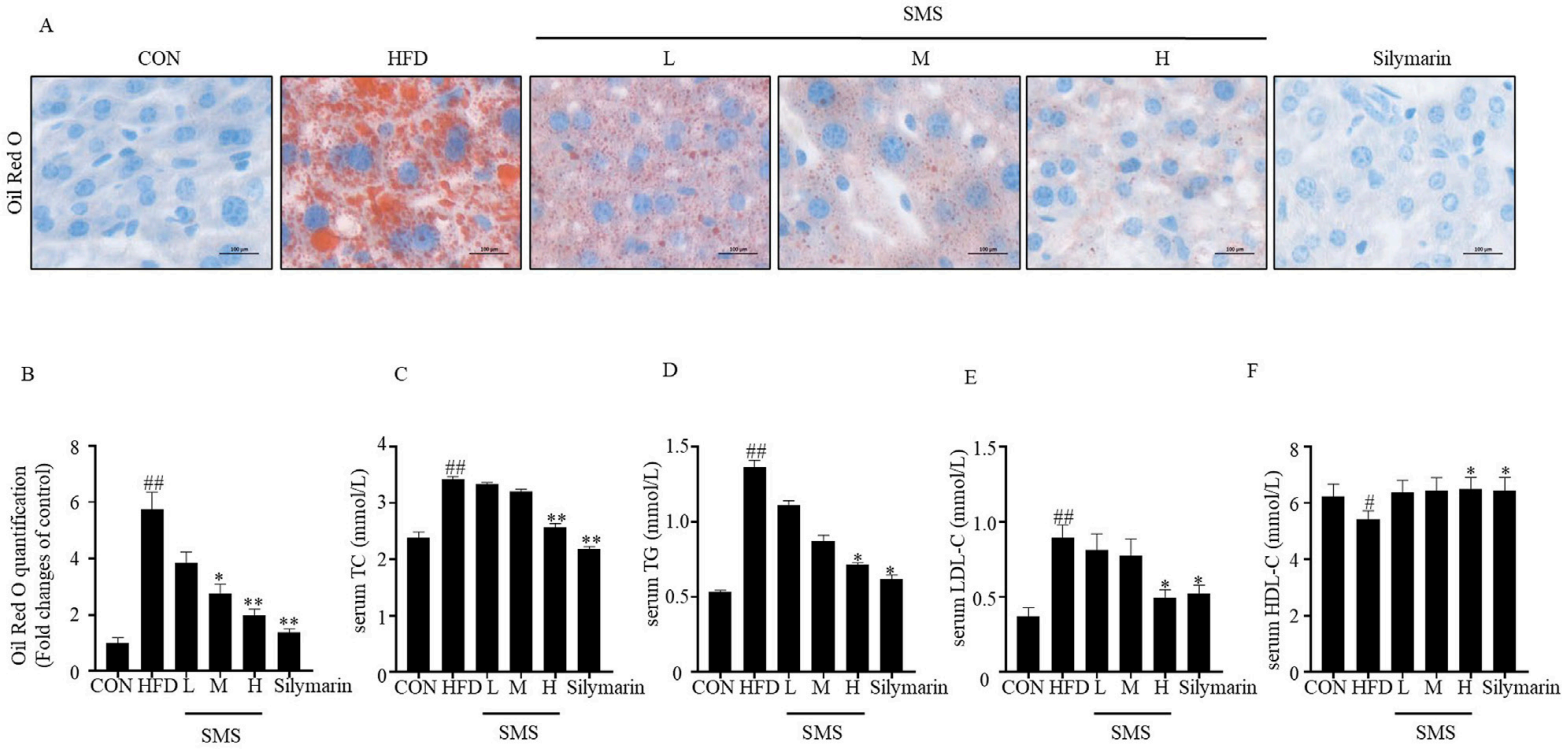
Effect of SMS on lipid accumulation of NAFLD in HFD-fed C57BL/6J mice. **(A)** Oil red O staining was performed on liver tissue sections of each mice. Representative images of one mice per group has been displayed by light microscope (400 ×). **(B)** The quantification statistical result of Oil Red O staining. The levels of serum **(C)** TC, **(D)** TG, **(E)** LDL-C and **(F)** HDL-C were detected by reagent kits. Silymarin (100 mg/kg) was used as the positive control. Results are mean ± SD (n = 10). ^#^ *P* < 0.05, ^##^ *P* < 0.01 vs. CON group; ^*^ *P* < 0.05, ^**^ *P* < 0.01 vs. HFD group.

Lipid accumulation plays a significant role in the initiation and progression of liver injury in NAFLD. Oil Red O staining demonstrated a notable increase in orange lipid droplets in the group subjected to HFD. Subsequently, the abundance of these lipid droplets decreased upon treatment with varying concentrations of SMS. The quantitative analysis of Oil Red O staining results indicated a significant reduction in orange lipid droplets with increasing drug concentration (Figures 3A,B). In addition, SMS treatment effectively inhibited the levels of TC, TG, and LDL-C in H group compared to HFD (Figures 3C–E). Furthermore, SMS treatment led to an elevation in HDL-C levels (Figure 3F). In conclusion, SMS administration alleviated hepatic lipid deposition *in vivo*.

### 3.4 SMS inhibit oxidative stress and inflammation of NAFLD in HFD fed C57BL/6J mice

**FIGURE 4:**
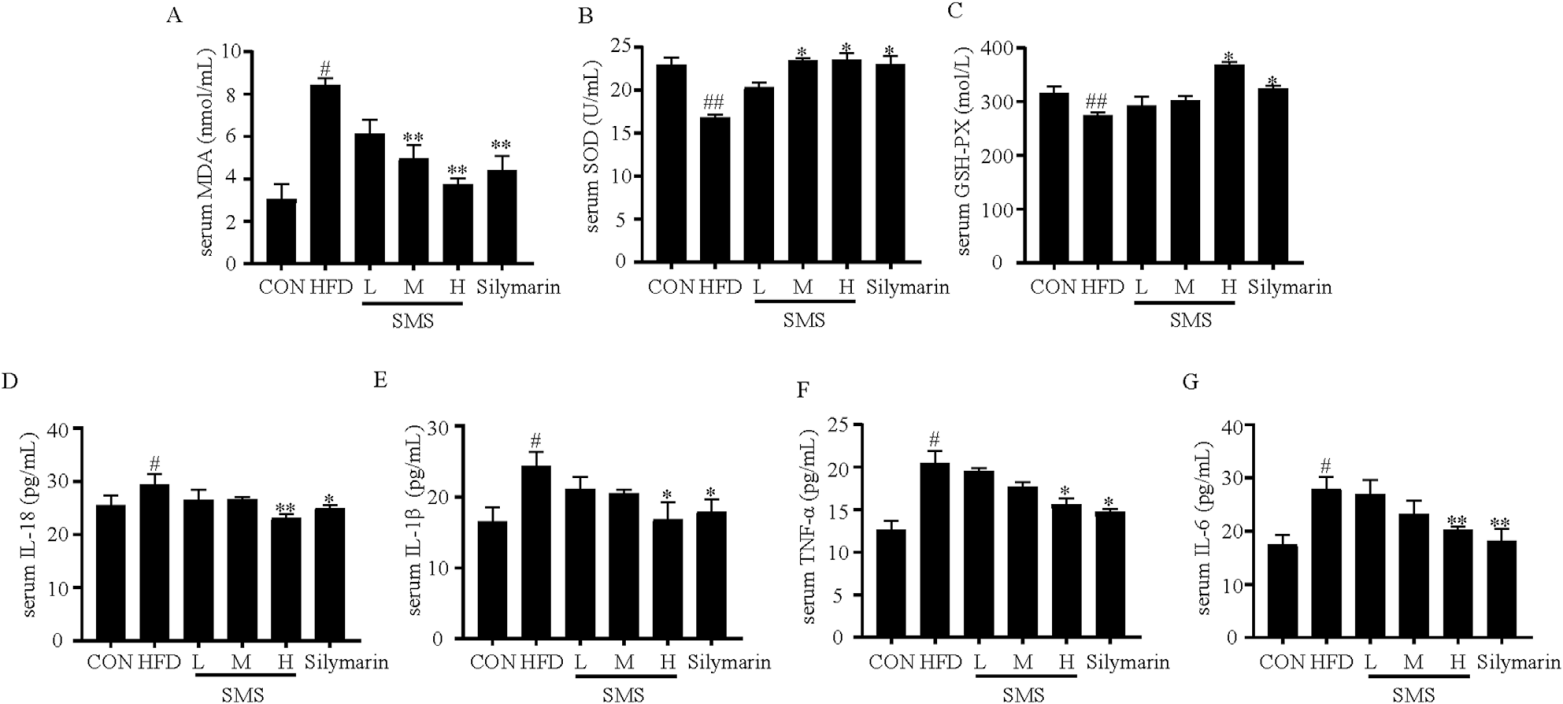
Effects of SMS on oxidative stress and inflammation of NAFLD in HFD-fed C57BL/6J mice. The levels of serum **(A)** MDA, **(B)** SOD, **(C)** GSH-Px, **(D)** IL-18, **(E)** IL-1β, **(F)** TNF-α and **(G)** IL-6 were determined by reagent kits. Were determined by reagent kits. Silymarin (100 mg/kg) was used as the positive control. Results are mean ± SD (n = 10). ^#^ *P* < 0.05, ^##^ *P* < 0.01 vs. CON group; ^*^ *P* < 0.05, ^**^ *P* < 0.01 vs. HFD group.

Oxidative stress and inflammation play a significant role in both the onset and progression of liver injury in NAFLD (Li J. et al., 2021). The MDA activity increased in the HFD group and diminished after SMS treatment. Furthermore, the levels of SOD and GSH-Px exhibited a notable rise compared to the HFD group (Figures 4A–C). Compared with HFD group, SMS treatment resulted in dose-dependent inhibition of expression levels of TNF-α, IL-6, IL-18, and IL-1β, with significant improvement in group H (Figures 4D–G). In conclusion, these findings indicate that SMS mitigates liver oxidative stress and inflammation *in vivo*.

### 3.5 SMS inhibit pyroptosis via TXNIP/NLRP3 pathway in HFD fed C57BL/6J mice

**FIGURE 5:**
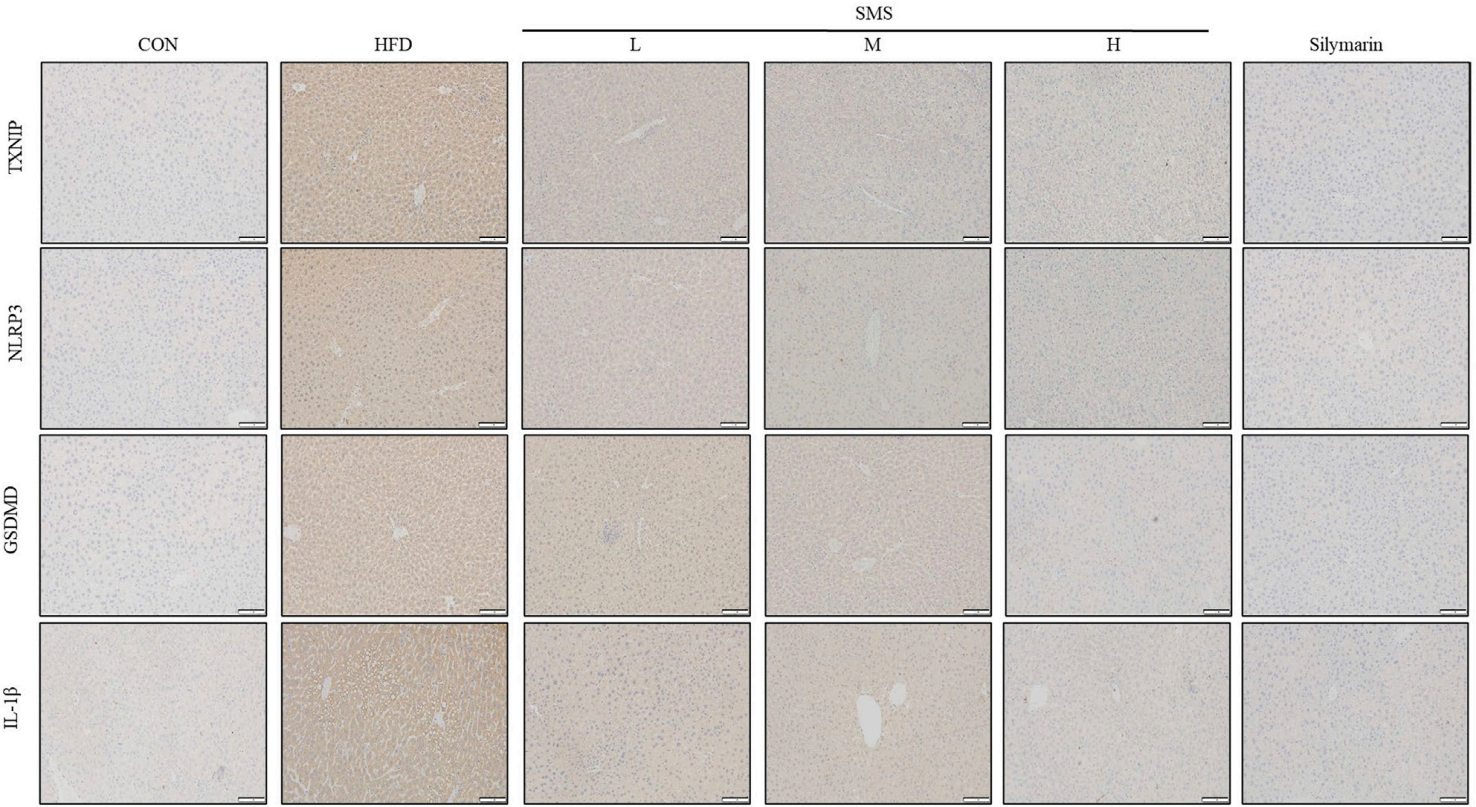
Effect of SMS on TXNIP/NLRP3 pathway of pyroptosis in HFD-fed C57BL/6J mice. Snap frozen liver tissue sections of mice were analyzed by IHC for TXNIP, NLRP3, GSDMD and IL-1β and a representative image of mice per group has been displayed (200×). Silymarin (100 mg/kg) was used as the positive control.

The impact of SMS on pyroptosis was assessed by analyzing snap-frozen liver tissue sections from mice via IHC for TXNIP, NLRP3, GSDMD, and IL-1β (Figure 5). The staining indicated that SMS treatment led to a dose-dependent reduction in the levels of TXNIP, NLRP3, GSDMD, and IL-1β compared to the HFD group. Overall, the SMS extract suppresses pyroptosis in non-alcoholic fatty liver disease through the TXNIP/NLRP3 pathway.

### 3.6 SMS alleviate lipid deposition in FFA induced HepG2 cells

**FIGURE 6:**
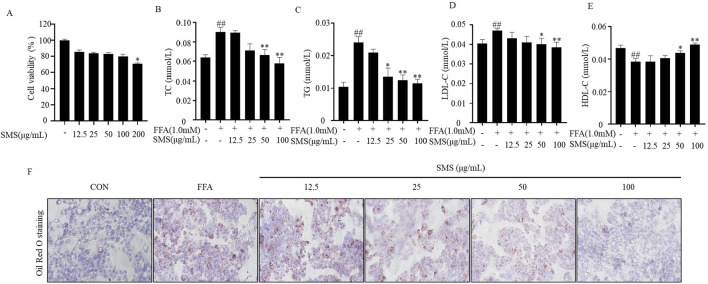
Effects of SMS on lipid accumulation in FFA induced HepG2 cells. Cells were treated with different concentrations of SMS (12.5 μg/mL-100 μg/mL) and then induced with FFA (1 mM) for 24 h each. **(A)** Cell viability was evaluated by MTT assay. The levels of **(B)** TC, **(C) **TG, **(D)** LDL-C and **(E)** HDL-C were detected by reagent kits. **(F)** Oil red O staining was performed on HepG2 cells and displayed by light microscope (200×). Results are mean ± SD. (n = 3) ^#^ *P* < 0.05, ^##^ *P* < 0.01 vs. CON group; ^*^ *P* < 0.05, ^**^ *P* < 0.01 vs. FFA group.

In cell experiments, SMS (200 μg/mL) significantly inhibited cell proliferation (Figure 6A). However, SMS at lower concentrations had no effect on cell proliferation. Consequently, subsequent studies will use concentrations of SMS ranging from 12.5 to 100 μg/mL. Established *in vitro* lipid deposition cell culture model using FFA treatment in HepG2 cells FFA treatment group. The results showed that significantly elevated expression levels of TG, TC, and LDL-C in the FFA group were reversed by SMS in a dose-dependent manner, and concentrations of 50 and 100 μg/mL were significantly improved (Figures 6B–D). Additionally, treatment with SMS increased levels of HDL-C (Figure 6E). Oil Red O staining showed that there were obvious orange lipid droplets increased in the FFA treatment group compared to the CON group (Figure 6F). However, orange lipid droplets were decreased after treated with various concentrations SMS. Taken together, these results indicate that SMS reduces lipid deposition *in vitro*.

### 3.7 SMS alleviate liver oxidative stress and inflammation in FFA induced HepG2 cells

**FIGURE 7:**
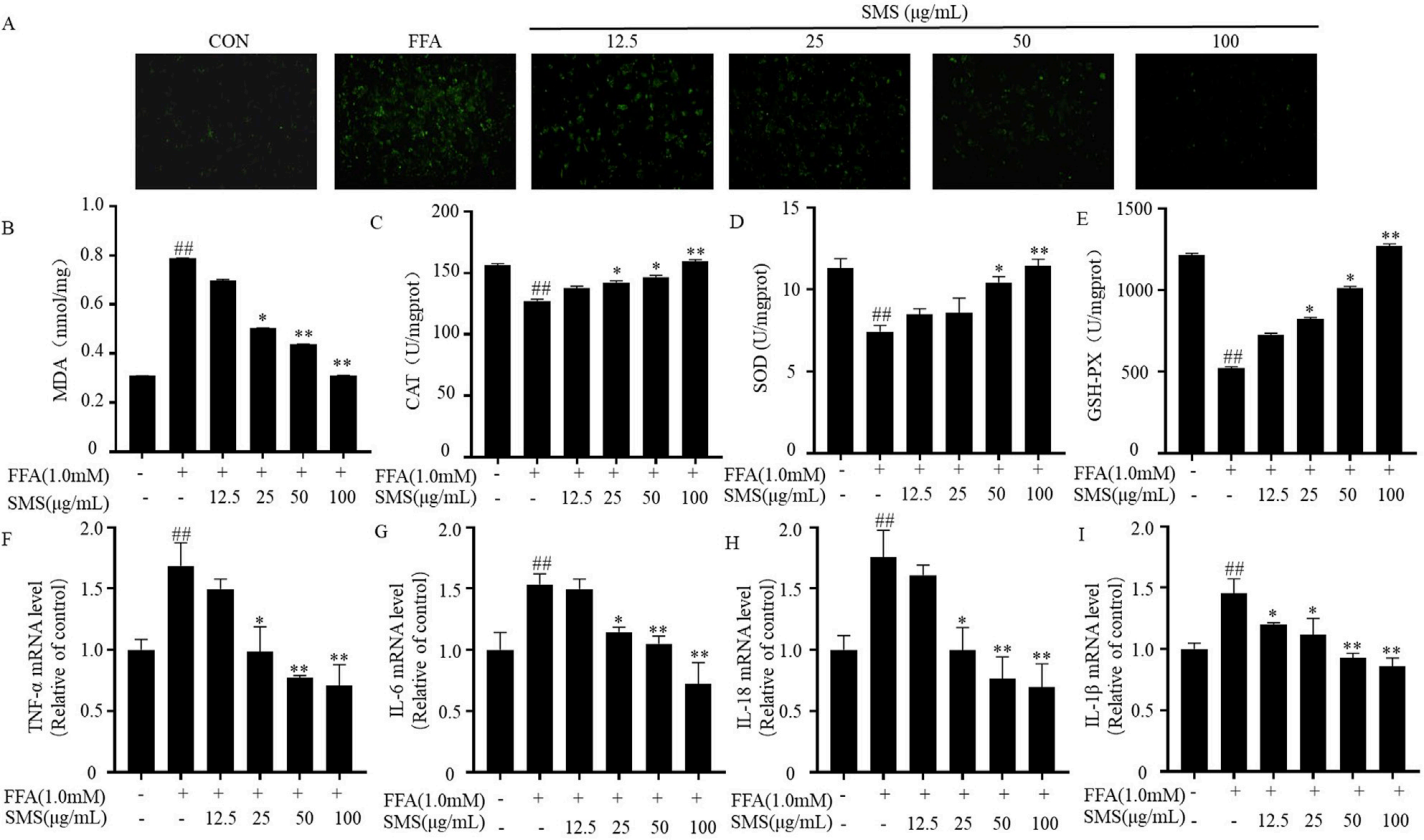
Effect of SMS on liver oxidative stress and inflammation in FFA induced HepG2 cells. Cells were treated with different concentrations of SMS (12.5 μg/mL-100 μg/mL) and then induced with FFA (1 mM) for 24 h each. **(A)** The fluorescence intensity of ROS was detected by immunofluorescence staining. The levels of **(B)** MDA, **(C)** CAT, **(D)** SOD and **(E)** GSH-Px were detected by reagent kits. The levels of **(F)** TNF-α, **(G)** IL-6, **(H)** IL-18 and **(I)** IL-1β mRNA expression were detected by qRT-PCR. Results are mean ± SD. (n = 3) ^#^ *P* < 0.05, ^##^ *P* < 0.01 vs. CON group; ^*^ *P* < 0.05, ^**^ *P* < 0.01 vs. FFA group.

To further confirm the antioxidative and anti-inflammatory effects of SMS. The antioxidative effect of SMS was assessed by evaluating intracellular ROS levels through fluorescence staining. Treatment with SMS following FFA induction significantly mitigated ROS levels in HepG2 cells (Figure 7A). Furthermore, the antioxidative impact of SMS was validated by evaluating the levels of the lipid peroxidation product (MDA) and antioxidant enzymes (CAT, SOD, and GSH-Px) using assay kits. Concordant with the intracellular ROS levels, MDA concentrations increased in the FFA group and decreased following SMS administration. Moreover, the levels of CAT, SOD, and GSH-Px were significantly elevated compared to the FFA group (Figures 7B–E). The anti-inflammatory effect of SMS was examined using qRT-PCR. Treatment with SMS in a dose-dependent manner suppressed the expression of TNF-α, IL-6, IL-18, and IL-1β mRNA in comparison to the FFA group (Figures 7F–I). Collectively, these findings demonstrate that SMS mitigates oxidative stress and inflammation *in vitro*.

### 3.8 SMS inhibit release of inflammatory factors in FFA induced HepG2 cells

**FIGURE 8:**
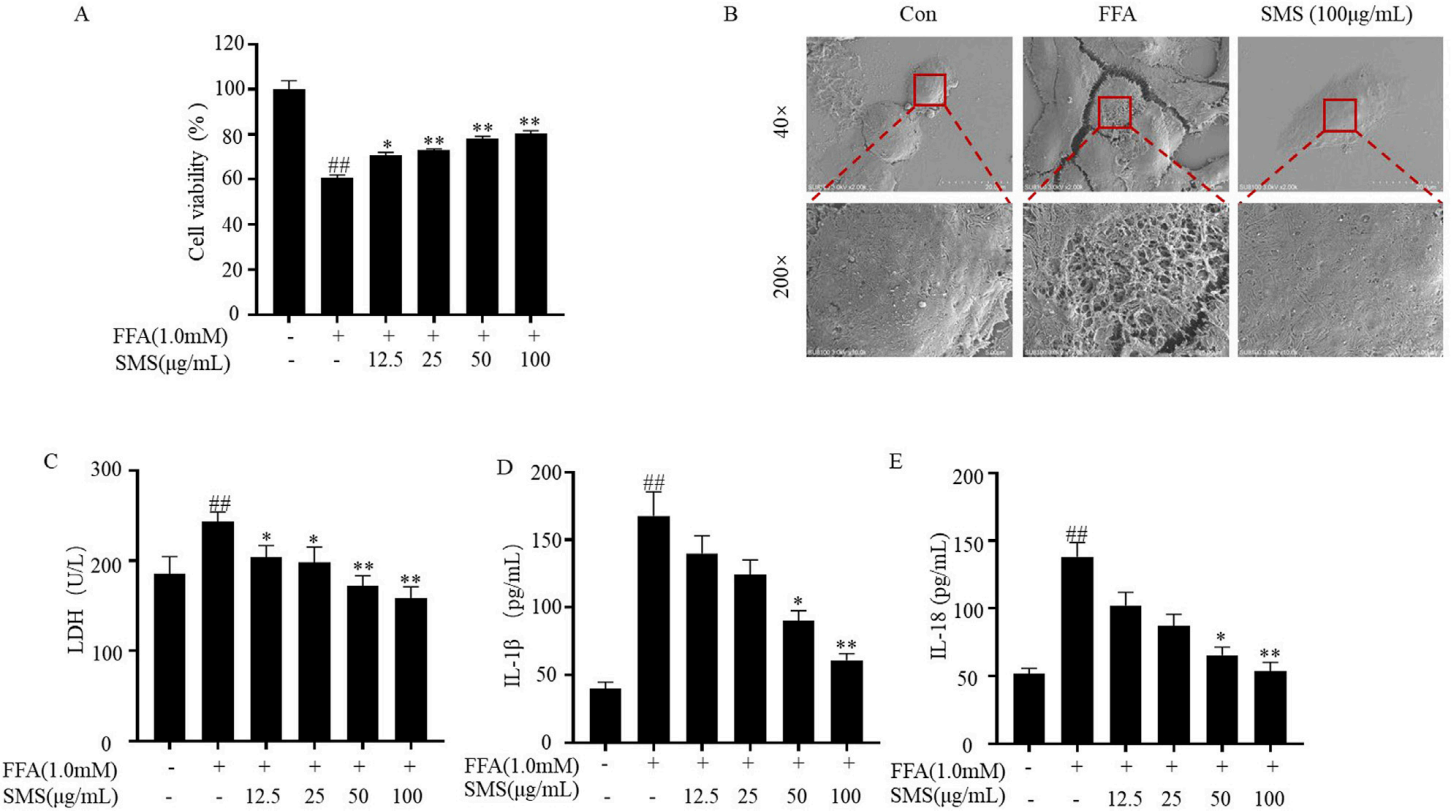
Effect of SMS on release of inflammatory factors in FFA induced HepG2 cells. Cells were treated with different concentrations of SMS (12.5 μg/mL-100 μg/mL) and then induced with FFA (1 mM) for 24 h each. **(A)** Cell viability was evaluated by MTT assay. **(B)** Representative SEM images (40 or 200×). The levels of **(C)** LDH, **(D)** IL-1β and **(E)** IL-18 were detected by reagent kits. Results are mean ± SD (n = 3). ^#^ *P* < 0.05, ^##^ *P* < 0.01 vs. CON group; ^*^ *P* < 0.05, ^**^ *P* < 0.01 vs. FFA group.

To determine the protective effect of SMS on cell injury induced by FFA. Cell viability significantly increased compared with FFA group (Figure 8A), indicating the protective effect of SMS on FFA-induced HepG2 cells. The morphology of FFA cultured HepG2 cells was compared using SEM. The results showed that the surface of cell membrane in the CON group remained intact. FFA group exhibited a lot of membrane pores, while the SMS (100 μg/mL) group showed a significant reduction in the number of membrane pores compared to the FFA group (Figure 8B). Assessed the effect of SMS on release of maker of pyroptosis (Lactate dehydrogenase, LDH) and inflammatory factors (IL-18, IL-1β). Treatment with SMS inhibited the levels of LDH, IL-18, and IL-1β compared to the FFA group in a dose-dependent manner (Figures 8C–E). Taken together, these results indicate that reduce the number of membrane pores and release of inflammatory factors *in vitro*.

### 3.9 SMS inhibit pyroptosis via TXNIP/NLRP3 pathway in FFA induced HepG2 cells

**FIGURE 9:**
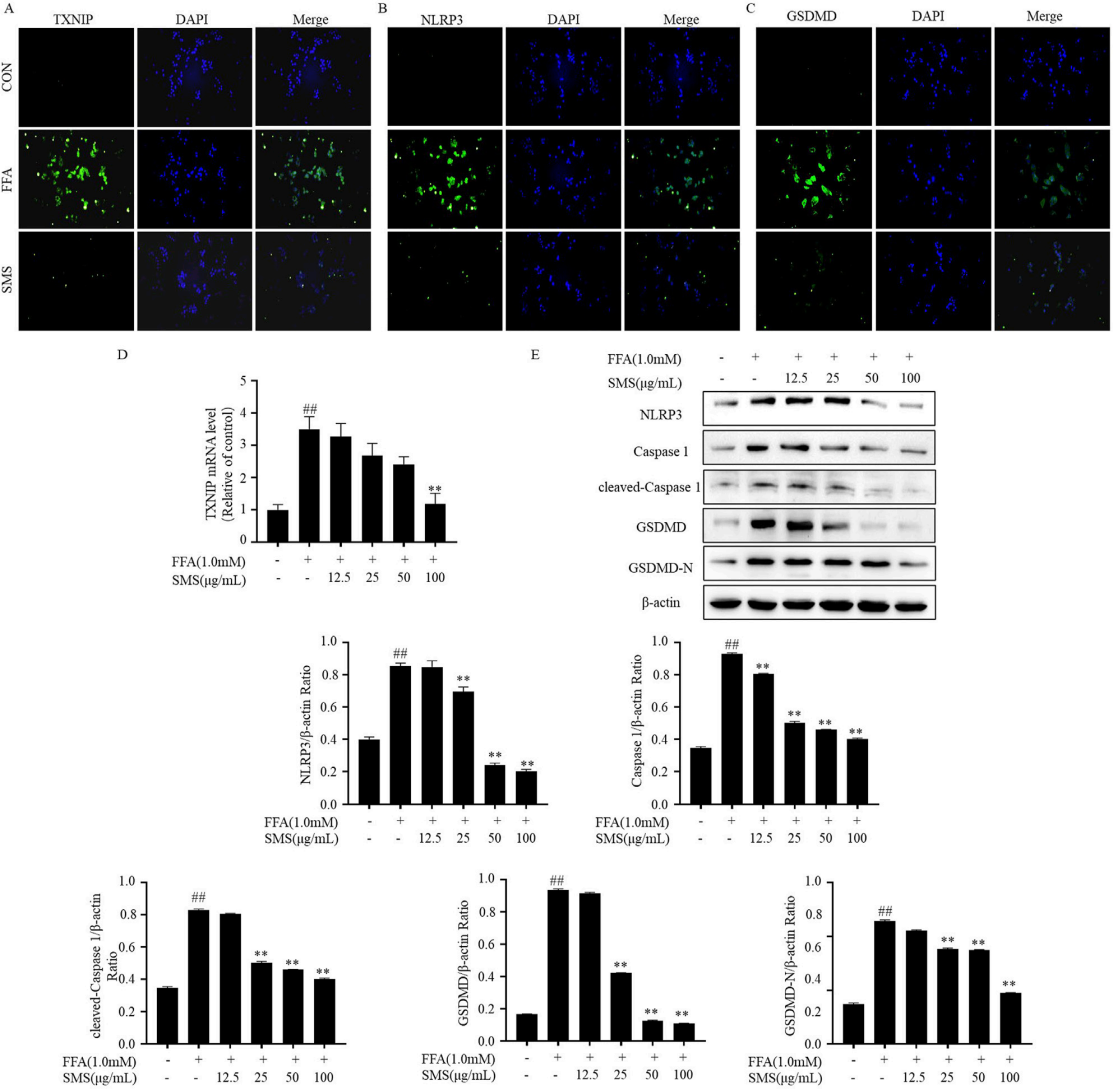
Effect of SMS on TXNIP/NLRP3 pathway in FFA induced HepG2 cells. Cells were treated with different concentrations of SMS and then induced with FFA (1 mM) for 24 h each. **(A–C)** The fluorescence intensity of TXNIP, NLRP3 and GSDMD was detected by immunofluorescence staining. The green color represented TXNIP, NLRP3 and GSDMD staining. **(D)** The levels of TXNIP mRNA expression were detected by qRT-PCR. **(E)** The expression of TXNIP, NLRP3, caspase 1, cleaved-caspase 1, GSDMD and GSDMD-N were detected by Western blotting, and their expression was normalized relative to β-actin. Results are mean ± SD (n = 3). ^#^ *P* < 0.05, ^##^ *P* < 0.01 vs. CON group; ^*^ *P* < 0.05, ^**^ *P* < 0.01 vs. FFA group.

To investigate the impact of SMS on pyroptosis, HepG2 cells induced by FFA were exposed to varying concentrations of SMS and subsequently subjected to immunofluorescence staining (Figures 9A–C). Representative images from each group confirmed that SMS alleviated NAFLD by inhibiting pyroptosis, the levels of TXNIP, NLRP3, and GSDMD proteins were upregulated, and this escalation was mitigated by SMS. qRT-PCR findings revealed a dose-dependent inhibition of TXNIP mRNA expression following SMS treatment compared to the FFA group (Figure 9D). Western blot analysis demonstrated that the markedly elevated expression levels of TXNIP, NLRP3, caspase 1, cleaved-caspase 1, and pyroptosis-related proteins in the FFA group were dose-dependently reversed by SMS (Figure 9E). In addition, studies on the active compounds of SMS have shown that four alkaloids can effectively reduce the expression of LDH and have a certain anti-pyroptosis effect (Supplementary Figure S1A–C). These results collectively indicate that SMS suppresses pyroptosis via the TXNIP/NLRP3 pathway *in vitro*.

### 3.10 SMS inhibits inflammatory damage by regulating pyroptosis mediated via TXNIP in FFA induced HepG2 cells

**FIGURE 10:**
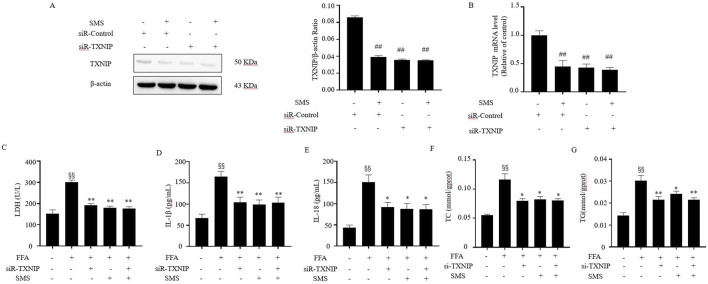
Effect of SMS on the inflammation in FFA induced TXNIP knockdown HepG2 cells. **(A)** The protein level of TXNIP were detected by Western blotting with siRNA-TXNIP. **(B)** Cells were treated with SMS (100 μg/mL) and then induced with FFA (1 mM) for 24 h each. The levels of TXNIP mRNA expression were determined by qRT-PCR. The levels of **(C)** LDH, **(D)** IL-1β, **(E)** IL-18, **(F)** TC and **(G)** TG were detected by reagent kits. Results are mean ± SD (n = 3). ^#^ *P* < 0.05, ^##^ *P* < 0.01 vs. siR-Control group; ^§^ *P* < 0.05, ^§§^ *P* < 0.01 vs. CON group; ^*^ *P* < 0.05, ^**^ *P* < 0.01 vs. FFA group.

HepG2 cells were subjected to TXNIP silencing using siRNA to assess its impact on pyroptosis. The findings exhibited successful delivery of siRNA-TXNIP into HepG2 cells, resulting in decreased levels of TXNIP mRNA and protein (Figures 10A–C). The silencing of TXNIP significantly reduced the expression of TC, TG, IL-18, and IL-1β in FFA-induced HepG2 cells compared to the Con group, and the therapeutic effect of SMS was abolished (Figures 10D,E). These outcomes indicate that SMS attenuates the expression of inflammatory markers and mitigates lipid accumulation by suppressing TXNIP expression.

## 4 Discussion

According to the Tibetan medicine classics such as “Four Medical Classics” (དཔལ་Ȳན་ȅད་བཞི།) and “Xin Xiu Jing Zhu Ben Cao” (༄༅། ། ཤེལ་གོང་ཤེལ་ེང་གསར་བȌིགས། །), *S. moorcroftiana* seeds have traditional uses in treating jaundice, hepatitis, purulent tonsillitis, diphtheria, and parasitic diseases. These seeds have been found to possess inhibitory effects on liver fibrosis (Min et al., 2018), protective effects against acute pneumonia (Xu et al., 2023), and anti-parasitic effects (Luo Y. et al., 2018). In our previous research, SMS increased the viability of t-BHP-induced HepG2 cells and may play a protective role in liver by inhibiting oxidative stress through MAPK pathway (Yuan et al., 2022).

In our previous research, sophocarpine, matrine, oxymatrine, and oxysophocarpine were isolated and identified from *S. moorcroftiana* extract (Yuan et al., 2016). The presence of alkaloid components was additionally verified using LC-MS (Yuan et al., 2022). This study involved quantifying the levels of sophocarpine, matrine, oxymatrine, and oxysophocarpine through HPLC analysis (Figures 1A,B). Previous studies have potential benefits of these compounds: sophocarpine for improving lipid metabolism and reducing inflammation and steatosis (Song et al., 2011; Zhang et al., 2020), matrine for significantly mitigating liver inflammation and lipid peroxidation by modulating endoplasmic reticulum stress and restoring mitochondrial function (Gao et al., 2018), oxymatrine for ameliorating liver fat accumulation in NAFLD models (Zhang et al., 2020), and oxysophocarpine exhibits effects in improving oxidative stress and inflammation factors in mice with colitis (Liu et al., 2023).

NAFLD is a common chronic liver disease and a serious pathological process characterized by fat accumulation in the liver, leading to NASH or hepatocellular carcinoma (Bedossa, 2017; Friedman et al., 2018). The pathological progression of NAFLD is closely related to lipid accumulation, oxidative stress and inflammation (Cobbina and Akhlaghi, 2017; Chen et al., 2020). NAFLD is caused by increased accumulation of TC and TG in the liver (Wang et al., 2021) Silymarin can regulate energy metabolism, attenuate liver damage, and improve liver histology in NAFLD patients (Li et al., 2024) Currently, T2M drugs are mainly used in the treatment of NAFLD, including Liraglutide, Semaglutide, Dapagliflozin (Rong et al., 2023). The levels of serum TC, TG, ALT, and AST confirm the establishment of the model groups. TC, TG, LDL-C and HDL-C were decreased compare with HFD group (Figures 2A–F), And SMS effectively inhibits lipid accumulation. MDA is the most common marker of oxidative stress. NAFLD is accompanied by increased MDA activity and decreased antioxidant levels (CAT, SOD, and GSH-Px) (Korish and Arafah, 2013; Chen et al., 2020). This experiment suggests that SMS effectively improves NAFLD lesions by inhibiting oxidative stress, reducing MDA activity, and increasing the levels of SOD and GSH-Px (Figures 4A–C), Inflammation factors such as TNF-α and IL-1β play a crucial role in the pathogenesis of NAFLD (Mirea et al., 2018; Vachliotis and Polyzos, 2023). SMS decreased the expression levels of inflammatory factors such as TNF-α, IL-6, IL-18, and IL-1β in HFD-fed C57BL/6J mice (Figures 4D–G).

The lipid accumulation model induced by FFA in HepG2 cells is commonly utilized for NAFLD research *in vitro* (Park et al., 2019). In comparison to the control group, treatment with SMS significantly decreased TC, TG, and LDL-C levels while increasing high-density lipoprotein cholesterol HDL-C levels (Figures 6B–E), indicating a reduction in lipid accumulation. Moreover, SMS treatment led to lower levels of ROS, MDA, CAT, SOD, and GSH-Px, suggesting a decline in oxidative stress (Figures 7A–E). Additionally, SMS treatment resulted in decreased levels of TNF-α, IL-6, IL-18, and IL-1β, indicating a reduction in inflammation (Figures 7F–I). This study demonstrates that SMS can effectively reduce lipid accumulation, oxidative stress, and inflammation in both NAFLD mice and cellular models of lipid accumulation.

Pyroptosis represents a distinct type of programmed cell death, characterized by membrane pores that lead to cell rupture, in contrast to apoptosis, which does not involve the cell membrane (Wang et al., 2020). Pyroptosis, gut microbiota, autophagy, apoptosis are the cause of NAFLD (Lian et al., 2020; Huang et al., 2023). The administration of SMS resulted in a reduction in the number of membrane pores (Figure 8B). LDH expression levels are commonly utilized to assess pyroptosis (Xu et al., 2021), and following SMS treatment, LDH levels were diminished. Pyroptosis in triggering the release of pro-inflammatory cytokines as part of the innate immune response (Xu et al., 2018). The study findings indicate that SMS decreased the expression levels of inflammatory markers such as TNF-α, IL-6, IL-18, and IL-1β *in vitro*, with consistent results observed *in vivo* studies (Figures 7F–I, 4E–H).

GSDMD is a protein that drives the execution of pyroptosis. Upon activation, GSDMD is cleaved into GSDMD-N fragments, which subsequently create pores in the cell membrane (Li S. et al., 2021). This cascade triggers the release of inflammatory factors, ultimately resulting in cell death. The activation of GSDMD is intricately linked with the activation of the NLRP3 inflammasome (Zheng and Kanneganti, 2020; Yan et al., 2021), crucial for caspase 1-dependent pyroptosis. NLRP3 activation enhances caspase 1 cleavage by facilitating interactions between ASC and NLRP3 inflammasome domains (Aachoui et al., 2013; Elliott and Sutterwala, 2015; Sun et al., 2019). Furthermore, it promotes the maturation of IL-1β and IL-18, initiating an inflammatory response (Yoshihara et al., 2014). Cleaved caspase 1 targets GSDMD, resulting in GSDMD-N translocation to the inner leaflet of the plasma membrane, where oligomerization occurs, forming membrane pores (Liu et al., 2022). In this study, SMS was shown to inhibit NLRP3 inflammasome activation, reduce NLRP3 protein levels, and attenuate caspase 1 activity in free fatty acid-treated HepG2 cells and high-fat diet-fed mice livers. In summary, the findings suggest that SMS can suppress NLRP3/GSDMD protein expression, potentially ameliorating NAFLD by inhibiting pyroptosis.

TXNIP is a widely distributed protein that acts as an inhibitor of TRX, an oxidoreductase responsible for reducing oxidized proteins and scavenging free radicals. In individuals with diabetes or prediabetes, TXNIP expression is elevated (Muoio, 2007). Moreover, increased TXNIP expression is observed in NAFLD (Zheng et al., 2018; Guo et al., 2022). The release of TXNIP from TRX triggers the activation of NLRP3 (Zhou et al., 2010). Following siRNA-TXNIP treatment, a notable decrease in inflammatory factors and lipid accumulation levels, along with reduced LDH expression levels, was observed, suggesting that TXNIP could be a promising target for SMS in combating free fatty acid-induced pyroptosis in HepG2 cells. ROS significantly contribute to the pathogenesis of NAFLD, exacerbating its progression. ROS have long been recognized as key players in various cell death modalities, including pyroptosis, apoptosis, and necroptosis (Anania et al., 2018; Farzanegi et al., 2019). ROS generation can oxidize TRX, causing the dissociation of TXNIP from TRX (Zhou et al., 2010). Notably, experiments revealed that treatment with SMS markedly reduced ROS levels in FFA-induced HepG2 cells impacting TXNIP expression *in vitro* (Figure 3A).

**FIGURE 11:**
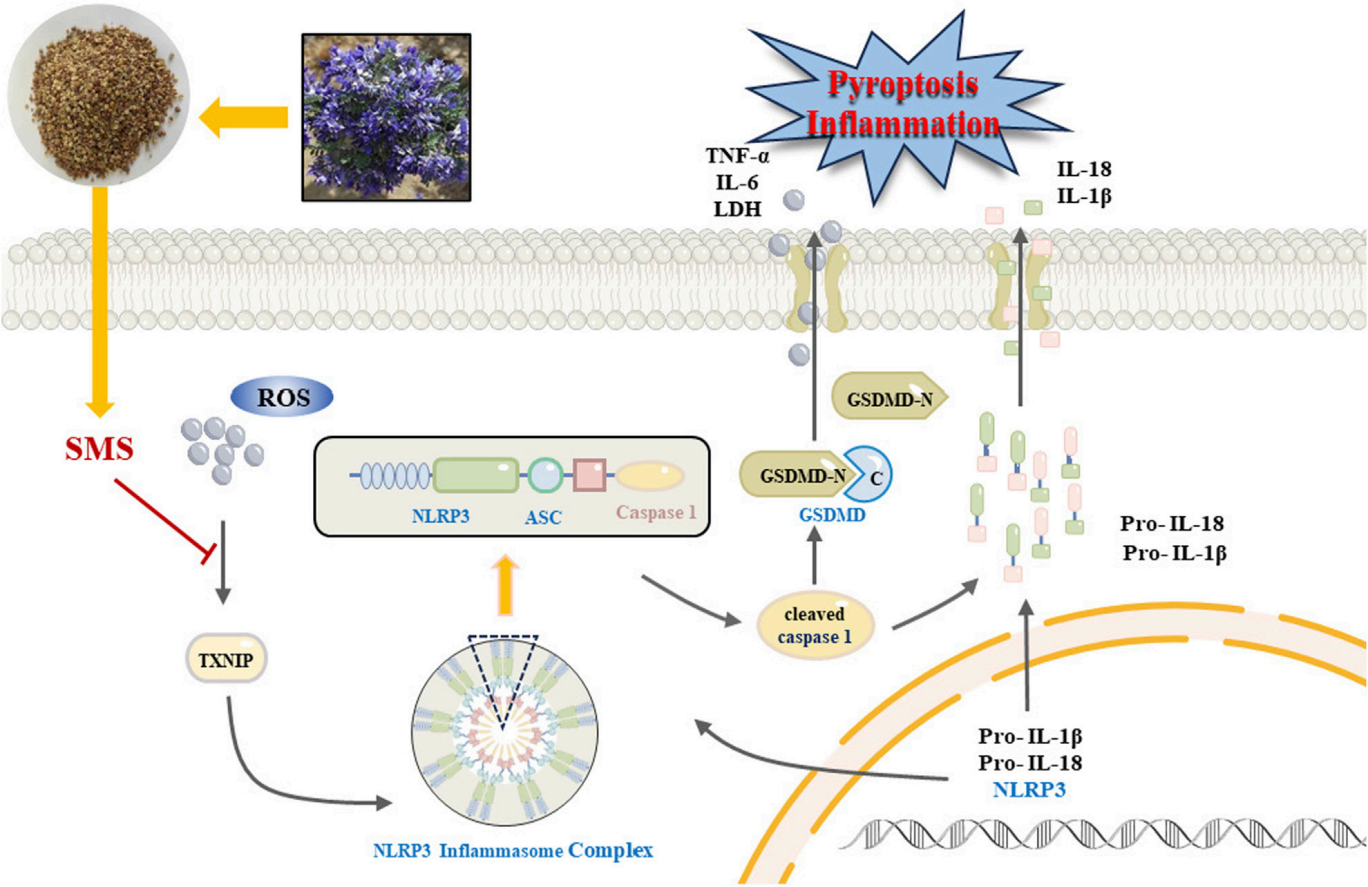
A schematic diagram representing that SMS *via* regulates NLRP3 inflammasome activation and pyroptosis *via* ROS/TXNIP pathway contributes to amelioration NAFLD.

In conclusion, our research findings indicate that SMS alleviates lipid accumulation, oxidative stress, and inflammation in NAFLD by inhibiting the ROS/TXNIP/NLRP3-mediated pyroptosis pathway (Figure 11). Thus, SMS could offer novel insights and approaches for NAFLD treatment. *S. moorcroftiana* showed potential value in the treatment of NAFLD, which can transform into NASH and liver fibrosis, and further studies on liver diseases will be conducted in subsequent studies.

## Data Availability

The original contributions presented in the study are included in the article/Supplementary Material, further inquiries can be directed to the corresponding authors.

## Glossary

AKP: Alkaline phosphatase
ALT: Alanine aminotransferase
ASC: Apoptosis associated speck-like protein containing a caspase recruitment domain
AST: Aspartate aminotransferase
CAT: Catalase
DMSO: Dimethyl sulfoxide
ECL: Enhanced chemiluminescence kit
ELISA: Enzyme-linked immunosorbent assay
FBS: Fetal bovine serum
FFA: Free fatty acid
GSDMD: Gasdermin D
GSH-PX: Glutathione peroxidase
H&E staining: Hematoxylin eosin staining
HDL-C: High-density lipoprotein cholesterol
HPLC: High performance liquid chromatography
IgG: Immunoglobulin G
IL-18: Interleukin-18
IL-1β: Interleukin-1β
LDH: Lactate dehydrogenase
LDL-C: Low-density lipoprotein cholesterol
MDA: Malondialdehyde
MEM: Minimum essential medium
MTT: 3-[4,5-dimethylthiazol-2-yl]-2,5-diphenyltetrazolium bromide
NAFLD: Non-Alcoholic Fatty Liver Diseas
NLRP3: NOD-like receptor thermal protein domain associated protein 3
OD: Optical density
P/S: Penicillin/streptomycin
PMSF: Phenylmethanesulfonyl fluoride
qRT-PCR: Quantitative real-time polymerase chain reaction
RIPA: Radioimmunoprecipitation lysis buffer
ROS: Reactive oxygen species
SD: Standard deviation
SDS-PAGE: Sodium dodecyl sulfate-polyacrylamide gel electrophoresis
SEM: Scanning electron microscopy
siRNA: Small interfering RNA
SMS: 70% ethanol extract of *S. moocroftiana* seeds
SOD: Superoxide dismutase
TC: Total cholesterol
TG: Triglyceride
TNF-α: Tumor necrosis factor-α
TRX: Thioredoxin
TXNIP: Thioredoxin-interacting protein
WB: Western blotting
