# Supplementary material for: Sophora moocroftiana seeds ethanol extract regulates NLRP3 inflammasome activation and pyroptosis via ROS/TXNIP pathway to amelioration of NAFLD in vitro and in vivo
Source: Front Pharmacol. 2025 Aug 25;16:1622178. doi: 10.3389/fphar.2025.1622178 (PMC12415560; doi:10.3389/fphar.2025.1622178)
Supplement: Supplementary file 1 [file DataSheet1.docx]

Supplementary Material


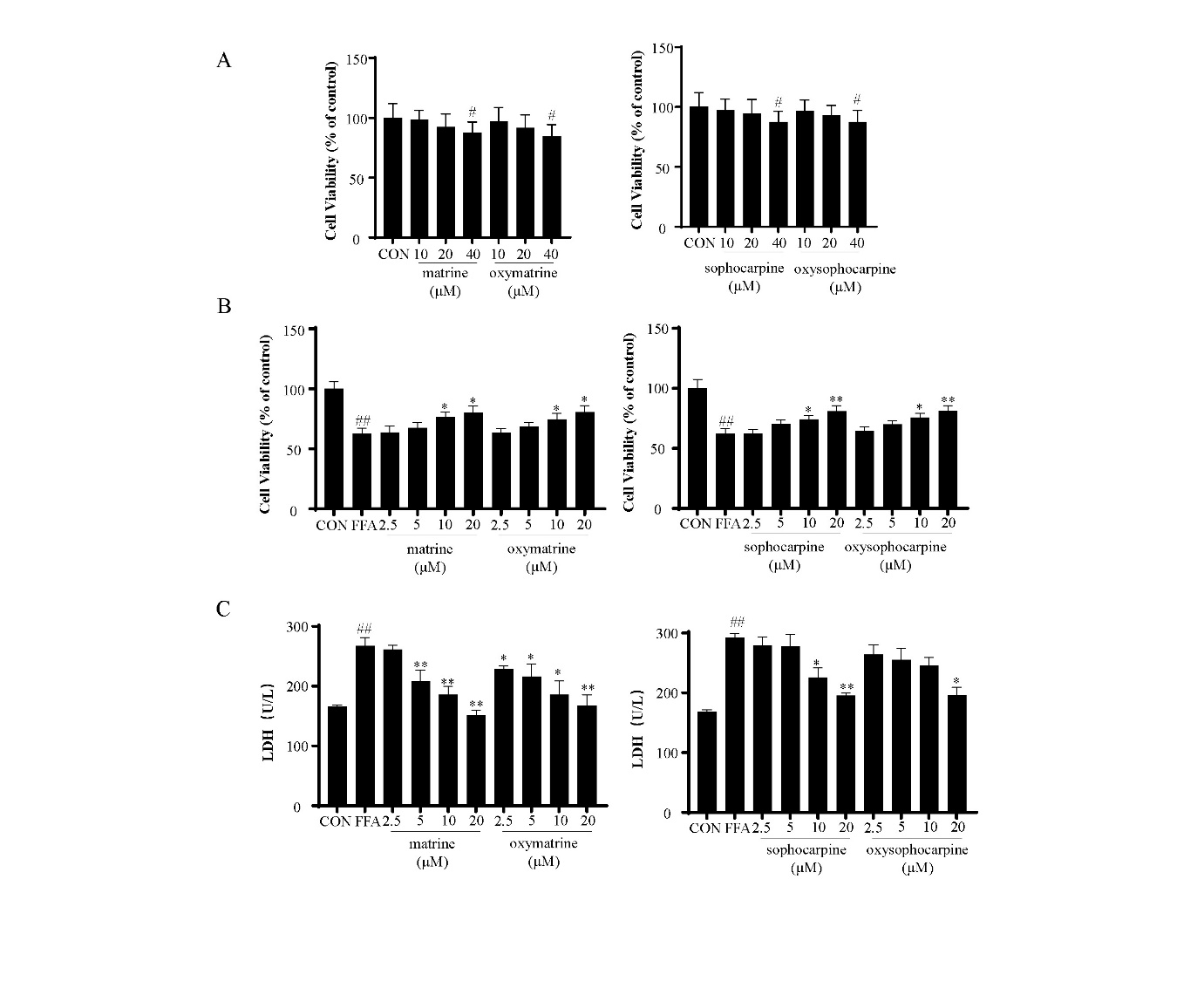


Figure S1. Effect of Matrine, Sophocarpine, Oxysophocarpine and Oxymatrine on release of inflammatory factors in FFA induced HepG2 cells. Cells were treated with different concentrations of Matrine, Sophocarpine, Oxysophocarpine and Oxymatrine and then induced with FFA (1mM) for 24 h each. Cell viability (A) and cytoprotection (B) were evaluated by MTT assay. The levels of (C)LDH were detected by reagent kits. Results are mean ± SD (n =3). ^#^*P*<0.05, ^##^*P*<0.01 vs. CON group; ^*^*P*<0.05, ^**^*P*<0.01 vs. FFA group.
